# Prediction of subsequent fragility fractures: application of machine learning

**DOI:** 10.1186/s12891-024-07559-y

**Published:** 2024-06-04

**Authors:** Mozhdeh Zabihiyeganeh, Alireza Mirzaei, Pouria Tabrizian, Aryan Rezaee, Abbas Sheikhtaheri, Azade Amini Kadijani, Bahare Amini Kadijani, Ali Sharifi Kia

**Affiliations:** 1 Bone and Joint Reconstruction Research Center, Department of Orthopedics, School of Medicine, University of Medical Sciences, Baharestan Sq, Tehran, Iran; 2 Department of Orthopaedic Surgery, University of Minnesota, Minneapolis, MN USA; 3 Student Research Committee, School of Medicine, Iran University of Medical Sciences, Tehran, Iran; 4 Department of Health Information Management, School of Health Management and Information Sciences, Iran University of Medical Sciences, Tehran, Iran; 5 Department of Medical Physics, Faculty of Medical Sciences, Tarbiat Modares University, Tehran, Iran; 6 Department of Computer Science, Faculty of Science, Western University, London, ON Canada

**Keywords:** Osteoporosis, Fragility fracture, Subsequent fracture, Machine learning, Data mining

## Abstract

**Background:**

Machine learning (ML) has shown exceptional promise in various domains of medical research. However, its application in predicting subsequent fragility fractures is still largely unknown. In this study, we aim to evaluate the predictive power of different ML algorithms in this area and identify key features associated with the risk of subsequent fragility fractures in osteoporotic patients.

**Methods:**

We retrospectively analyzed data from patients presented with fragility fractures at our Fracture Liaison Service, categorizing them into index fragility fracture (*n* = 905) and subsequent fragility fracture groups (*n* = 195). We independently trained ML models using 27 features for both male and female cohorts. The algorithms tested include Random Forest, XGBoost, CatBoost, Logistic Regression, LightGBM, AdaBoost, Multi-Layer Perceptron, and Support Vector Machine. Model performance was evaluated through 10-fold cross-validation.

**Results:**

The CatBoost model outperformed other models, achieving 87% accuracy and an AUC of 0.951 for females, and 93.4% accuracy with an AUC of 0.990 for males. The most significant predictors for females included age, serum C-reactive protein (CRP), 25(OH)D, creatinine, blood urea nitrogen (BUN), parathyroid hormone (PTH), femoral neck Z-score, menopause age, number of pregnancies, phosphorus, calcium, and body mass index (BMI); for males, the predictors were serum CRP, femoral neck T-score, PTH, hip T-score, BMI, BUN, creatinine, alkaline phosphatase, and spinal Z-score.

**Conclusion:**

ML models, especially CatBoost, offer a valuable approach for predicting subsequent fragility fractures in osteoporotic patients. These models hold the potential to enhance clinical decision-making by supporting the development of personalized preventative strategies.

**Supplementary Information:**

The online version contains supplementary material available at 10.1186/s12891-024-07559-y.

## Background

Osteoporosis represents a significant public health concern within the aging population [1, 2]. Epidemiological data suggest that approximately one-third of women and one-fifth of men over the age of 50 will experience at least one osteoporotic fracture in their lifetime [3]. The incidence of such fractures is estimated to increase almost two folds by 2045 [4]. Patients with a history of fragility fracture face an elevated risk of subsequent fractures, linked to increased morbidity, mortality, and diminished quality of life [5, 6], thereby necessitating prevention of a subsequent fracture.

The identification of risk factors for subsequent fragility fractures is a crucial element in preventing re-fracture [7]. Prior research has identified numerous predictors, including age, gender, the site of the initial fracture, and comorbid conditions like hypertension and diabetes [6, 8–11]. Despite the recognized importance of these factors in preventing further fractures, they are often overlooked in clinical decision-making due to a lack of personalized risk assessment tools [12].

The World Health Organization developed the Fracture Risk Assessment Tool (FRAX) to evaluate the 10-year probability of bone fractures due to osteoporosis using clinical risk factors [13]. Despite being a significant advancement in fracture risk assessment, FRAX has several limitations, including but not limited to not taking into account changes in risk factors over time and providing a static risk assessment [14].

In response to these limitations, there have been significant strides in applying machine learning (ML) in personalized medicine [15, 16], including the prediction of cancer recurrence [17, 18], to enhance osteoporosis management. Numerous studies have employed a variety of ML techniques such as logistic regression, XGBoost, random forest, K-nearest neighbor, support vector machine, decision trees, and neural networks. These methods address various facets of osteoporosis from risk prediction and early detection to diagnosis, treatment, and management [19–23].

The potential of ML to predict re-fracture risk in osteoporotic patients remains largely untapped. A predictive ML model could facilitate personalized preventative strategies encompassing structured exercise, fall prevention, nutritional supplementation, custom orthoses, and prophylactic pharmacotherapy [24]. This study aims to develop an ML-based model to predict the risk of subsequent fragility fractures in patients with a history of such fractures, incorporating clinically relevant features.

## Methods

### Data sources and study population

This retrospective analysis received approval from the institutional review board of our institute, designated by the code IR.IUMS.REC.1401.106, which granted a waiver for informed consent. This study involved patients presenting with fragility fractures at the FLS of Shafa Orthopedic Hospital, affiliated with the Iran University of Medical Sciences in Tehran, from 2020 to 2023. The cohort was categorized into two groups: those with an initial fragility fracture (*n* = 905) and those with a subsequent fragility fracture (*n* = 195). The index fragility fractures were located in the distal radius (38%), lumbar spine (18%), femoral neck (15%), proximal humerus (5%), and other locations (24%). The re-fractures were mainly located in the distal radius (47%), femoral neck (32%), proximal humerus (14%), and other locations (7%). The mean time interval between the primary and secondary fragility fracture was 41.2 ± 31.7 months (range 1-120).

Re-fractures were mainly self-reported. However, the clinical history of patients was checked by the involved rheumatologist to make sure it was a subsequent osteoporotic fracture and not a traumatic fracture.

Inclusion criteria were those that were regarded for FLS (age ≥ 50 years and osteoporosis-related fractures). Any fracture caused by low-trauma fracture, often following a fall from standing height or less, was considered an osteoporotic fracture, excluding fractures at the toes, metatarsal bones, fingers, metacarpal bones, skull, facial bones, and mandible [25].

In total, 1100 patients who were registered during the study period were included in the analysis. Input features were extracted as an Excel file from the data captured by the FLS system. We excluded features considered irrelevant to the osteoporotic fracture based on the earlier evidence [26–30] and physician opinion. Features with more than 30% missing values or more than 95% of the data distributed in one class were excluded. In total, 118 features were identified at initial inspection, of which 27 features met the study criteria and were used for training the models. Since the FLS database in our center is grounded upon the workup of the causes of secondary osteoporosis, factors such as ESR, CRP, PTH, 25(OH)D, ALP, etc. which could indicate a secondary root of osteoporosis, were included in the feature sets.

Model training was done for males and females separately, considering the exclusion of pregnancy frequency and menopause age in the male group. As a result, model training in the male group was performed with 25 features. Characteristics of these features are demonstrated in detail in Table 1.

**Table 1:** Patients’ characteristics

Variable	Female patients *n* = 712 ( 65%)	Male patients *n* = 388 (35%)	Female Patients’ data Missing percentage	Male Patients’ data Missing percentage
**Age (year)**	64.6 ± 9.5	62.5 ± 9.6	0	0
**Age of Menopause (year)**	47.9 ± 5.6	-	6.18	-
**BMI (kg/m** ^**2**^ **)**	28.8 ± 4.8	26.2 ± 4	1.54	1.03
**Re-fracture**				
**Yes** **No**	149 [21] 563 (79)	46 (11.9) 292 (88.1)	0	0
**Femoral Neck BMD (g/cm** ^**2**^ **)**	0.638 ± 0.176	0.698 ± 0.125	8.43	11.34
**Femoral Neck T-score**	-1.92 ± 1.1	-1.57 ± 1	8.43	11.34
**Femoral Neck Z-score**	-0.5 (-4.4 to 2.8)	-0.5 (-2.8 to 3.1)	8.43	11.34
**Total Hip BMD (g/cm** ^**2**^ **)**	0.821 ± 0.164	0.910 ± 0.169	8.01	12.37
**Total Hip T-score**	-0.9 (-4.8 to 2.8)	-0.6 (-4 to 5)	8.29	12.63
**Total Hip Z-score**	0.2 (-4.1 to 3.5)	0.1 (-2.9 to 10)	8.15	12.37
**Spine BMD (g/cm** ^**2**^ **)**	0.804 ± 0155	0.873 ± 0.159	10.53	11.86
**Spine T-Score**	-2.1 ± 1.4	-1.77 ± 1.44	10.25	12.11
**Spine Z-Score**	-0.530 ± 1.3	-0.863 ± 1.56	10.25	12.37
**Serum CRP (mg/dL)**	7 (1 to 130)	9 (0 to 120)	3.51	3.35
**Serum ALP (U/L)**	192.6 ± 65.9	183.9 ± 60	4.78	4.64
**Serum BUN (mg/dl)**	16.9 ± 6.1	17.1 ± 5.4	5.06	4.38
**Serum Creatinine (mg/dl)**	0.95 ± 0.19	1 ± 0.2	3.51	3.35
**Parathyroid hormone (ng/L)**	52.4 ± 25	49.6 ± 17.4	17.13	17.01
**25(OH)D (gr/ dl)**	36.8 ± 14.5	33.3 ± 13.1	7.44	9.79
**Serum calcium (mg/dl)**	8.9 ± 0.6	8.6 ± 0.8	4.35	5.15
**Serum phosphor (mg/dl)**	3.7 ± 0.7	3.5 ± 0.8	5.20	5.67
**Pregnancy numbers**				
**0** **1** **2** **3** **4** **5** **More than 5**	44 (6.2) 35 (4.9) 102 (14.3) 112 (15.7) 119 (16.7) 98 (13.8) 202 (28.4)	-	13.62	-
**Anticoagulant consumption**				
**Yes** **No**	42 (5.9) 670 (94.1)	21 (5.4) 367 (94.6)	17.56	16.75
**Current smoker**				
**Yes** **No**	39 (5.4) 693 (94.6)	157 (40.5) 231 (59.5)	21.77	11.86
**History of smoking**				
**Yes** **No**	36 (5.1) 696 (94.9)	28 (7.2) 360 (92.8)	5.20	4.12
**Using calcium supplement**				
**Yes** **No**	97 (13.6) 615 (86.4)	29 (7.5) 359 (92.5)	5.48	3.61
**Diabetes mellitus**				
**Yes** **No**	151 (21.3) 561 (78.7)	60 (15.5) 328 (84.5)	16.15	16.75

Quantitative variables are demonstrated with mean ± standard deviation for normally distributed quantitative parameters, with median (range) for non-normally distributed quantitative parameters, and with numbers (%) for qualitative parameters.

### Data preprocessing

Outliers in the dataset were identified as data points lying beyond ± 3 standard deviations from the mean of a given feature. These outliers were subsequently replaced with the nearest values within the interquartile range boundaries. Numerical data underwent normalization to scale the values, while categorical variables were transformed via one-hot encoding, assigning 1 for “Yes” and 0 for “No.”

The rate of missing data for the male dataset varied from 1.03 to 17.01%, and for the female dataset, it ranged from 1.54 to 21.77%. For normally distributed numerical variables, the mean of the feature was used to impute missing values. In contrast, the median was employed for skewed numerical data. The mode was used for imputing missing categorical data, chosen based on the most frequent value within each class (re-fracture or no re-fracture). Detailed missing data rates for each feature are tabulated in Table 1.

### Features and feature selection

The primary outcome, subsequent fragility fracture, was recorded as a binary variable (yes/no). The dataset comprised 26 features, excluding the target variable. These features encompassed demographics (age, sex, menopause age, BMI), laboratory results (CRP, ALP, serum Vitamin D, PTH), medical history (comorbidities, medication use), and densitometry measurements (BMD, T-score, Z-score).

Seven distinct feature sets were engineered to predict fragility in both genders. Six of these were derived using recursive feature elimination with cross-validation (RFECV) applied to random forest, XGBoost, CatBoost, logistic regression, LightGBM, and AdaBoost algorithms. The seventh set was manually selected based on prior evidence and clinician expertise, deemed relevant for predicting future fragility risk.

### Data balancing

Initial models, based on features selected by physician opinion and trained using the XGBoost algorithm, demonstrated suboptimal performance (AUC = 0.502 for females and AUC = 0.498 for males), likely due to an imbalance in re-fracture instances. To address this, the synthetic minority oversampling technique (SMOTE) was implemented to augment the underrepresented class (re-fracture) in the datasets [31].

### Model Development, evaluation, and explainability

**Fig. 1:**
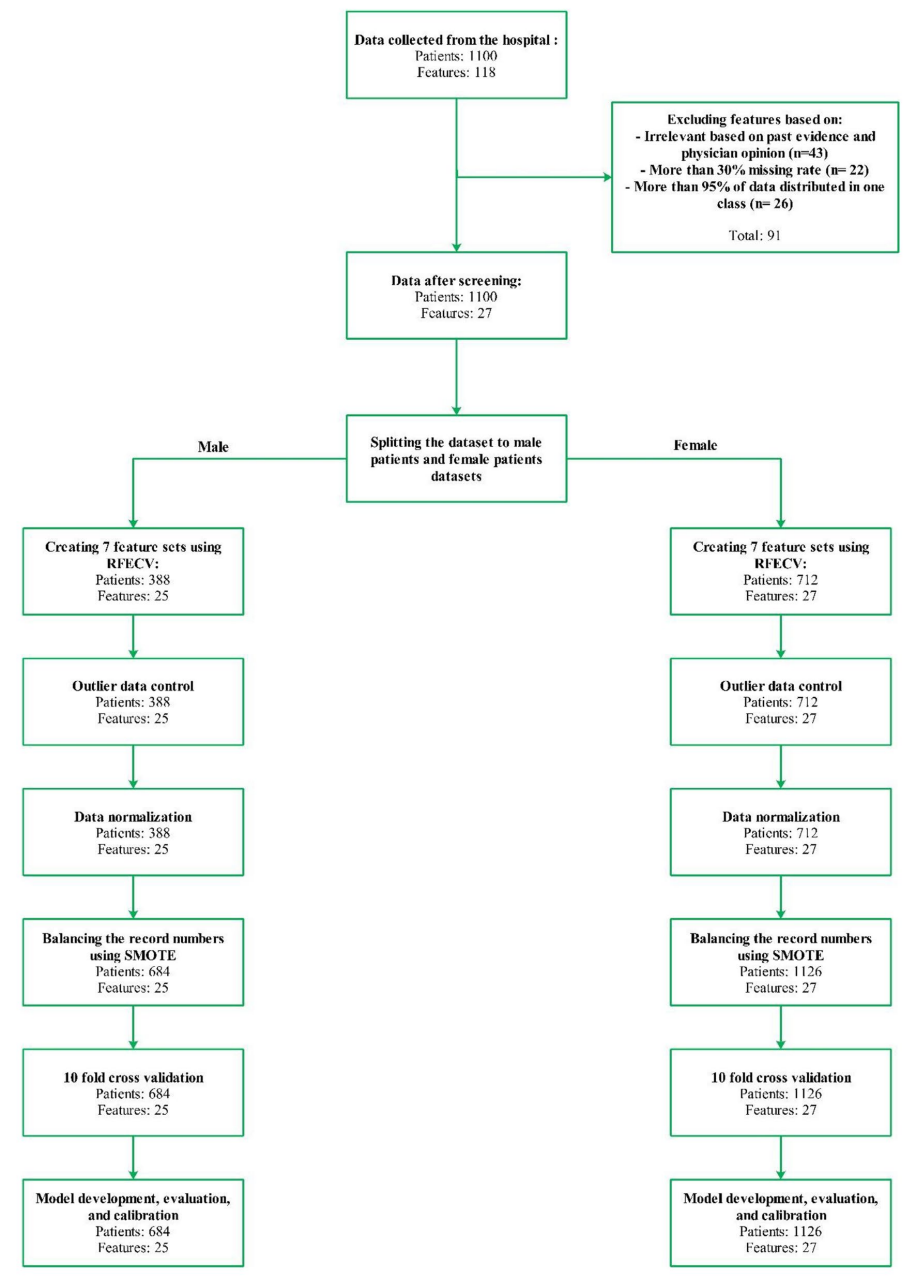
Study flow diagram

We employed an array of models for development, including random forest, XGBoost, CatBoost, logistic regression, LightGBM, AdaBoost, MLP, and SVM, utilizing 10-fold cross-validation as illustrated in Fig. 1. Hyperparameter optimization for these models was conducted using a variable grid for each algorithm in combination with GridSearchCV from the scikit-learn library.

Model performance was assessed using accuracy, the area under the receiver operating characteristic curve (AUC ROC), precision, recall, F1 score, logistic loss, and Brier score. Model comparison hinged on the F1 score and accuracy, leading to the selection of the optimal models for both male and female patient groups. The contribution of individual features to the model performance was determined using Shapley Additive Explanations (SHAP) [32].

## Results

### Feature selection

Tables S1 and S2 present the details of the feature sets created using the male and female patients’ dataset.

## Model performance and evaluation

A summarized evaluation of the performance of various predictive models for female patients, using feature sets one through seven, is provided in Tables S3-S9. Generally, the CatBoost algorithm demonstrated superior performance across the majority of feature sets, with the exception of feature set 5, where the LightGBM algorithm was more effective. Logistic regression exhibited the least robust performance across all feature sets, with the exception of feature set 7, where the SVM model was the least effective.

The performance details of the predictive models for male patients across different feature sets are documented in Tables S10-S16. The CatBoost algorithm consistently outperformed the other models across all feature sets. Logistic regression generally displayed the least favorable performance, except in feature sets 4, 5, and 7, where the SVM model showed the weakest results.

The optimal model for predicting subsequent fragility fractures in female patients was the CatBoost model trained on feature set 2, achieving an accuracy of 0.870 and an F1 score of 0.882. For male patients, the most effective model was the CatBoost trained on feature set 6, with an accuracy of 0.934 and an F1 score of 0.938. The performance metrics for the top five predictive models for female and male patients are presented in Tables 2 and 3, respectively.

**Table 2:** Top 5 female patients’ prediction models

Rank	Algorithm	Feature set		Parameters	Accuracy	AUC	Precision	Recall	F1 Score	Log Loss	Brier Score
1	CatBoost		2	depth = 10, l2_leaf_reg = 7, learning_rate = 0.1, n_estimators = 700	0.870	0.951	0.826	0.948	0.882	0.324	0.100
2	CatBoost		4	depth = 10, l2_leaf_reg = 1, learning_rate = 0.1, n_estimators = 600	0.868	0.956	0.819	0.950	0.879	0.322	0.100
3	CatBoost		1	depth = 10, l2_leaf_reg = 1, learning_rate = 0.03, n_estimators = 700	0.857	0.951	0.804	0.948	0.869	0.333	0.104
4	CatBoost		3	depth = 10, l2_leaf_reg = 1, learning_rate = 0.03, n_estimators = 600	0.853	0.940	0.805	0.936	0.865	0.354	0.111
5	CatBoost		6	depth = 10, l2_leaf_reg = 1, learning_rate = 0.1, n_estimators = 300	0.828	0.903	0.795	0.886	0.837	0.431	0.131

**Table 3:** Top 5 male patients’ prediction models

Rank	Algorithm	Feature set		Parameters	Accuracy	AUC	Precision	Recall	F1 Score	Log Loss	Brier Score
1	CatBoost		6	n_estimators = 600, depth = 10, l2_leaf_reg = 1, learning_rate = 0.03	0.934	0.990	0.895	0.985	0.938	0.175	0.051
2	CatBoost		2	n_estimators = 300, depth = 6, l2_leaf_reg = 1, learning_rate = 0.1	0.931	0.984	0.888	0.988	0.935	0.197	0.055
3	CatBoost		1	n_estimators = 300, depth = 10, l2_leaf_reg = 1, learning_rate = 0.03	0.928	0.987	0.885	0.985	0.932	0.202	0.058
4	XGBoost		2	n_estimators = 700, max_depth = 15, learning_rate = 0.01, colsample_bytree = 0.3	0.923	0.975	0.911	0.938	0.923	0.270	0.074
5	CatBoost		3	n_estimators = 300, depth = 6, l2_leaf_reg = 1, learning_rate = 0.1	0.921	0.986	0.874	0.985	0.926	0.196	0.058

### Feature importance

#### Female patient’s prediction model

**Fig. 2:**
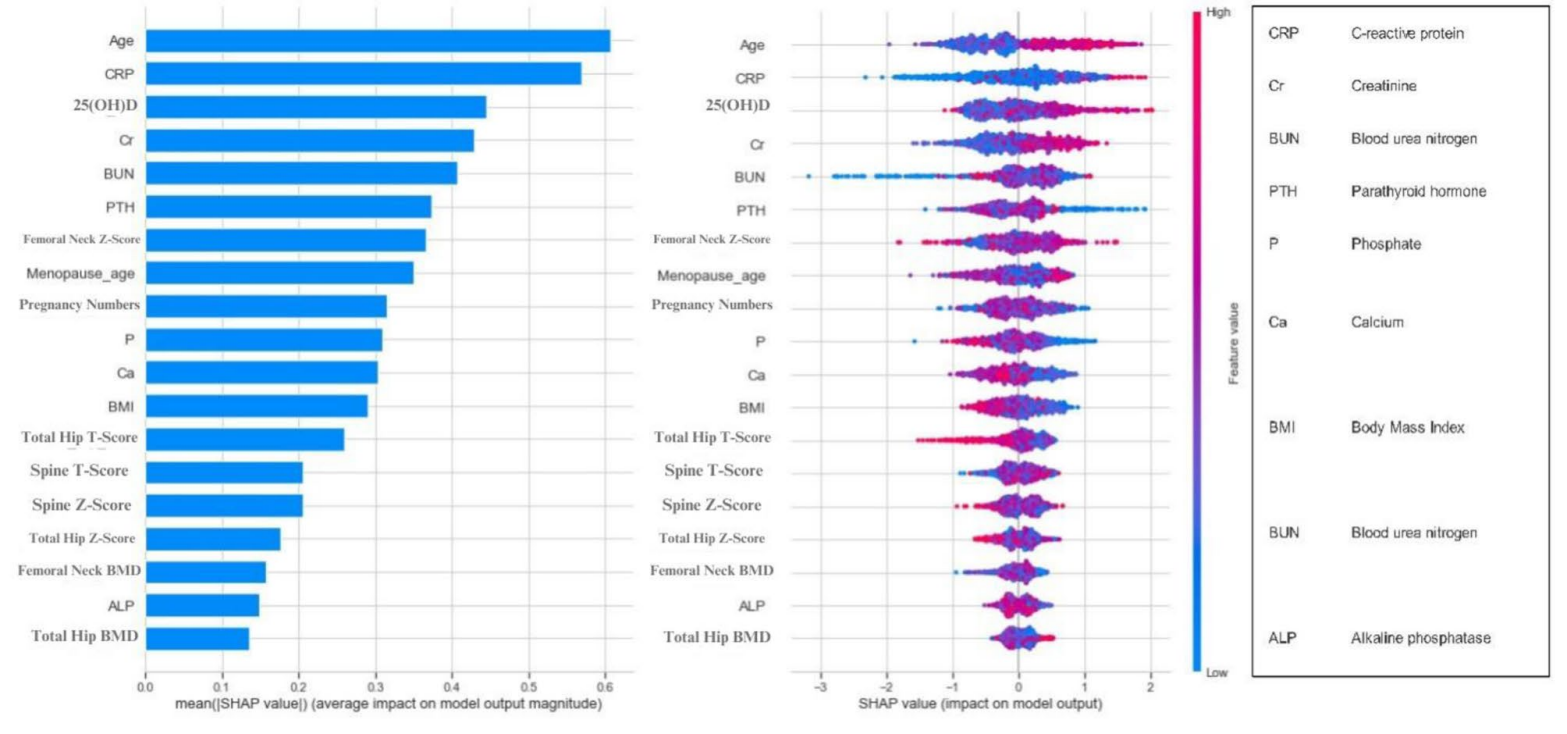
Shapley Additive Explanation (SHAP) feature importance for CatBoost prediction model in female patients

As depicted in Fig. 2, age, serum CRP, serum level of 25(OH)D (vitamin D3), serum creatinine, serum BUN, serum PTH, femoral neck Z-score, menopause age, number of pregnancies, serum phosphorus, serum calcium, and BMI had the highest contribution to the model’s prediction.

### Male patients’ prediction model

**Fig. 3:**
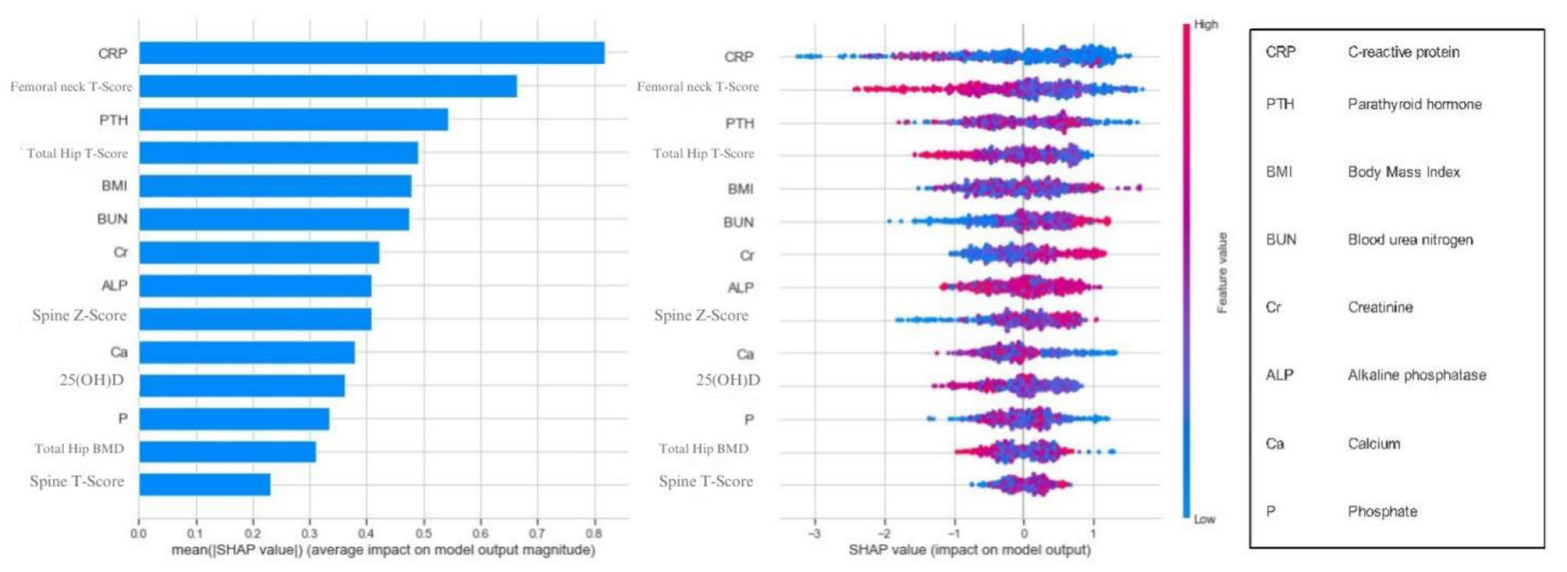
Shapley Additive Explanation (SHAP) feature importance for Male CatBoost prediction model in male patients

As presented in Fig. 3, serum CRP, femoral neck T-score, serum PTH, hip T-score, BMI, serum BUN, serum creatinine, serum ALP, and spinal Z-score had the highest amount of contribution to the model’s performance in order.

### Error analysis

#### Female patient’s prediction model

In total, there were 155 errors, of which 9 were false positives and 146 were false negatives. According to Figure S1, which presents the confusion matrix and heatmap of the error cases, ALP, PTH, 25(OH)D, age, menopause age, CRP, and BMI were more related to the error cases. As the color in the grid gets darker, it resembles a higher relation with errors.

### Male patient’s prediction model

Overall, there were 9 errors, which 6 were false negatives and 3 were false positives. As depicted in Figure S2, ALP, PTH, 25(OH)D, CRP, BUN, and BMI were most related to the error cases.

## Discussion

In this research, we assessed the predictive capabilities of various machine learning (ML) models in predicting subsequent fragility fractures within distinct male and female cohorts. Additionally, we identified the most contributing features in these predication models. For both genders, the CatBoost model emerged as the most accurate, yielding the highest predictive accuracy at 93.4% for males and 87% for females. The SHAP analysis revealed that in the female-specific models, the features that contributed most significantly included age, CRP, 25(OH)D, creatinine, BUN, PTH, femoral neck Z-score, menopause age, number of pregnancies, phosphorus, calcium, and BMI. For the male-specific models, the features with the greatest impact on the model’s predictive power were CRP, femoral neck T-score, PTH, hip T-score, BMI, BUN, creatinine, ALP, and spinal Z-score. To date, various studies have investigated the risk factors of re-fracture in osteoporotic patients sustaining a fragility fracture [6, 8–11]. Although these studies have provided valuable information, there is still a gap in the clinical application of this data, mainly due to the inability of physicians to interpret and implement these data in the process of treatment decision-making. ML algorithms are able to interpret this data according to the feature importance and provide a personalized risk for re-fracture, thereby translating the patients’ data into clinical practice [15, 16].

Following the advent of ML in medical sciences, the potential of these algorithms in osteoporosis management has been evaluated in many studies [33]. Although the use of ML algorithms in the prevention of subsequent fragility fractures has been considered, it has not received as much attention as it deserves. Shimizu et al. [34] evaluated the capability of ML algorithms for prediction and feature selection of re-fracture after surgical treatment of non-vertebral index fragility fracture. More than 7000 patients with an index fragility fracture were included in their study, randomly divided into training (75%) and test (25%) datasets. A decision-tree-based model (Light-GBM), Artificial Neural Network, and SVM model were developed for the prediction purpose. LightGBM model showed moderate accuracy for the prediction in the training (AUC = 0.90) and test dataset (AUC = 0.75), whereas the other models revealed poor performance (AUC < 0.60). Rheumatoid arthritis (RA) and chronic kidney disease (CKD) were the most relevant features for predicting the subsequent fracture. In the present study, we evaluated various ML models, including LightGBM and SVM. CatBoost was the most predictive ML model in our study, with a maximum AUC of 0.990 for the male group and 0.956 for the female group. However, the male and female populations were not evaluated separately in the study of Shimizu et al. Considering the smaller number of patients compared to the study of Shimizu et al., we used a cross-validation approach to test the performance of machine learning models. Features that had the highest contribution to the model’s prediction were significantly different from those reported by Shimizu et al., which could be attributed to the registration protocol. Since our center was a subspecialized orthopedic hospital, patients with RA, CKD, hyperthyroidism, and other important underlying disorders were not generally referred to our FLS department.

Ma et al. [35] compared the effectiveness of different ML algorithms in predicting new fractures after the treatment of index osteoporotic vertebral compression fractures. In a retrospective analysis of 529 patients, ML models including decision trees, random forests, SVM, gradient boosting machines (GBM), neural networks, regularized discriminant analysis (RDA), and logistic regression were compared in terms of their effectiveness in predicting new fractures occurring after surgical treatment of index fracture. The dataset was subdivided into the training (75%) and test set (25%). ML models were developed in training sets after ten cross-validations. Subsequently, the performance of each model was assessed in the test dataset. Almost all models predicted better than logistic regression, with random forest showing the maximum AUC (0.940). In contrast to the study of Ma et al., which was limited to the prediction of subsequent vertebral fragility fracture, the present study was not restricted by the location of the fragility fracture. Even so, both studies reveal the promising role of ML in the prediction of subsequent fragility fracture. The CatBoost algorithm, which was the best-predicting model in the present study, was not used in the study of Ma et al. Again, the male and female populations were not evaluated separately in the study of Ma et al.

Vries et al. [12] compared three ML algorithms, including the Cox regression, random survival forests (RSF), and an artificial neural network (ANN)-DeepSurv model, to design a risk assessment tool for future fractures. In total, 7578 patients with osteopenia or osteoporosis were included, of which 805 (11%) patients sustained a subsequent major osteoporotic fracture (MOF). For the complete dataset, including the osteopenia and osteoporosis patients, no significant difference was found between the discriminative ability of the three models. In the osteopenia group, the Cox regression model significantly outperformed the other models, with an AUC of 0.701 one year after the index fracture. Age, prior falls, simultaneous vertebral fracture, history of epilepsy, and age of menopause were independently associated with the incidence of subsequent MOF in the complete dataset using the Cox regression model. The predictive capability of the ML models used in the present study was remarkably higher than the study of Vries et al. This difference can be attributed to several factors, including the patient population, the type of fractures, or the ML model itself. These differences should be further investigated in future studies.

Regarding the feature importance, some features that were already acknowledged as predictors of fragility fracture, including age, sex, menopause age, and densitometry parameters, were found to be important features in our model’s development, as well. In addition, some features that were less frequently reported as predictors of subsequent fragility fracture in the general osteoporotic population were also included in our model’s development, including the CRP, BUN, and creatinine. High CRP levels, as a marker of chronic inflammation, have been earlier attributed to the increased risk of fragility fractures, although previous studies have yielded conflicting results [36, 37]. BUN and creatinine are acknowledged predictors of fragility fracture in osteoporotic patients with chronic kidney diseases, explained by the association between renal function and BMD [38–40]. However, these markers are rarely notified as predictors of fragility fractures in the general osteoporotic population, which could infer the power of ML algorithms to explore their predictive power.

Altogether, the results of the present study show that ML models could play an important role in the perdiction of subsequent fragility fractures. Therefore, optimization of these methods in the future could be regarded to empower clinicians to provide personalized re-fracture strategies. Such tools have already been designed for index fragility fractures (Fracture Risk Assessment Tool). However, the prevention of second re-fracture has received less attention and deserves more investigations in the future.

The present study had some strengths and weak points. The number of ML models evaluated in the present study was more than in earlier studies, and CatBoost, which was shown to be the most accurate model, was not used in earlier studies. Evaluation of the models separately for males and females could be the other strong point of the study, as menopause could be regarded as a confounding factor in males when models are trained on both sexes. The absence of an external validation set and a smaller number of patients, particularly in the male group, could be regarded as the weak points of this study. In addition, the study population was recruited from a subspecialized orthopedic hospital, and patients with important underlying disorders such as RA, CKD, hyperthyroidism, and other underlying disorders were not generally referred to our hospital. For this reason, the elaborated model might not be generalizable to other healthcare settings and patients with certain disorders.

## Conclusion

Machine learning (ML) models, and the CatBoost algorithm in particular, have demonstrated a strong ability to predict subsequent fragility fractures. As such, these models show promise as effective tools in predicting future fragility fractures in patients with osteoporosis. The further refinement and optimization of these ML models could aid clinicians in creating tailored prevention strategies to reduce the risk of future fragility fractures.

### Electronic supplementary material

Below is the link to the electronic supplementary material.

Supplementary Material 1

Supplementary Material 2

## Data Availability

No datasets were generated or analysed during the current study.
