# Supplementary material for: Prediction of subsequent fragility fractures: application of machine learning
Source: BMC Musculoskelet Disord. 2024 Jun 4;25:438. doi: 10.1186/s12891-024-07559-y (PMC11149176; doi:10.1186/s12891-024-07559-y)
Supplement: Supplementary file 1 — Supplementary Material 1 [file 12891_2024_7559_MOESM1_ESM.docx]

**Table of Figures**

[Table S1 Details of the feature sets created using the female patients' dataset. 2](#_Toc161331145)

[Table S2 Details of the feature sets created using male patients' dataset. 3](#_Toc161331146)

[Table S3 Performance of female patients' prediction models on feature set 1 4](#_Toc161331147)

[Table S4 Performance of female patients' prediction models on feature set 2 5](#_Toc161331148)

[Table S5 Performance of female patients' prediction models on feature set 3 6](#_Toc161331149)

[Table S6 Performance of female patients' prediction models on feature set 4 7](#_Toc161331150)

[Table S7 Performance of female patients' prediction models on feature set 5 8](#_Toc161331151)

[Table S8 Performance of female patients' prediction models on feature set 6 9](#_Toc161331152)

[Table S9 Performance of female patients' prediction models on feature set 7 10](#_Toc161331153)

[Table S10 Performance of male patients' prediction models on feature set 1 11](#_Toc161331154)

[Table S11 Performance of male patients' prediction models on feature set 2 12](#_Toc161331155)

[Table S12 Performance of male patients' prediction models on feature set 3 13](#_Toc161331156)

[Table S13 Performance of male patients' prediction models on feature set 4 14](#_Toc161331157)

[Table S14 Performance of male patients' prediction models on feature set 5 15](#_Toc161331158)

[Table S15 Performance of male patients' prediction models on feature set 6 16](#_Toc161331159)

[Table S16 Performance of male patients' prediction models on feature set 7 17](#_Toc161331160)

Table S1 Details of the feature sets created using the female patients' dataset.

| **Feature set** | **Method** | **Number of features** | **Features** |
| --- | --- | --- | --- |
| 1 | Physician opinion | 27 | 'Age', 'Menopause_age', 'Tscore_Hip_Total', 'CRP', 'Cr', 'ALP', 'BUN', 'P', 'Ca', 'PTH', 'Vit_D3', 'BMD_Spine', 'Tscore_Vertebra', 'Zscore_ Spine', 'BMD_Hip_Total', 'Zscore_Hip_Total', 'BMD_Femur_Neck', 'Tscore_Femur_Neck', 'Zscore_Femur_Neck', 'BMI', 'Pregnancy_Count', 'Histroy_Anticoagulant', 'Active_Smoking', 'History_Smoking', 'Calcium_Supplement', '‘History_of_diabetes’' |
| 2 | RFECV (Random Forest) | 19 | 'Age', 'Menopause_Age', 'Tscore_Hip_Total', 'CRP', 'Cr', 'ALP', 'BUN', 'P', 'Ca', 'PTH', 'Vit_D3', 'Tscore_ Spine', 'Zscore_ Spine', 'BMD_Hip_Total', 'Zscore_Hip_Total', 'BMD_Femur_Neck', 'Zscore_Femur_Neck', 'BMI', 'Pregnancy_Count' |
| 3 | RFECV (XGBoost) | 15 | 'Age', 'Menopause_Age', 'CRP', 'Cr', 'ALP', 'BUN', 'P', 'Ca', 'PTH', 'BMD_Femur_Neck', 'Zscore_Femur_Neck', 'Pregnancy_Count', 'Histroy_Anticoagulant', 'History_Smoking', '‘History_of_diabetes’' |
| 4 | RFECV (CatBoost) | 20 | 'Age', 'Menopause_Age', 'Tscore_Hip_Total', 'CRP', 'Cr', 'ALP', 'BUN', 'P', 'Ca', 'PTH', 'Vit_D3', 'BMD_ Spine', 'Tscore_ Spine', 'Zscore_ Spine', 'BMD_Hip_Total', 'Zscore_Hip_Total', 'BMD_Femur_Neck', 'Zscore_Femur_Neck', 'BMI', 'Pregnancy_Count' |
| 5 | RFECV (Logistic regression) | 4 | 'Cr', 'BMD_Hip_Total', 'Histroy_Anticoagulant', 'History_Smoking' |
| 6 | RFECV (LightGBM) | 6 | 'Menopause_Age', 'CRP', 'ALP', 'Ca', 'BMD_Femur_Neck', 'BMI' |
| 7 | RFECV (AdaBoost) | 9 | 'Age', 'Menopause_Age', 'Cr', 'ALP', 'Ca', 'Vit_D3', 'Tscore_ Spine', 'BMD_Femur_Neck', 'Pregnancy_Count' |

Table S2 Details of the feature sets created using male patients' dataset.

| **Feature set** | **Method** | **Number of features** | **Features** |
| --- | --- | --- | --- |
| 1 | Physician opinion | 25 | 'Age', 'Tscore_Hip_Total', 'CRP', 'Cr', 'ALP', 'BUN', 'P', 'Ca', 'PTH', 'Vit_D3', 'BMD_ Spine', 'Tscore_ Spine', 'Zscore_ Spine', 'BMD_Hip_Total', 'Zscore_Hip_Total', 'BMD_Femur_Neck', 'Tscore_Femur_Neck', 'Zscore_Femur_Neck', 'BMI', 'Histroy_Anticoagulant', 'Active_Smoking', 'History_Smoking', 'Calcium_Supplement', '‘History_of_diabetes’' |
| 2 | RFECV (Random Forest) | 12 | 'Age', 'Tscore_Hip_Total', 'CRP', 'Cr', 'ALP', 'BUN', 'Ca', 'PTH', 'Vit_D3', 'Zscore_ Spine', 'BMD_Hip_Total', 'Tscore_Femur_Neck' |
| 3 | RFECV (XGBoost) | 23 | 'Age', 'Tscore_Hip_Total', 'CRP', 'Cr', 'ALP', 'BUN', 'P', 'Ca', 'PTH', 'Vit_D3', 'BMD_ Spine', 'Tscore_ Spine', 'Zscore_ Spine', 'BMD_Hip_Total', 'Zscore_Hip_Total', 'BMD_Femur_Neck', 'Tscore_Femur_Neck', 'Zscore_Femur_Neck', 'Histroy_Anticoagulant', 'Active_Smoking', 'History_Smoking', 'Calcium_Supplement', '‘History_of_diabetes’' |
| 4 | RFECV (CatBoost) | 15 | 'Age', 'Tscore_Hip_Total', 'CRP', 'Cr', 'ALP', 'BUN', 'P', 'Ca', 'PTH', 'Vit_D3', 'BMD_Hip_total', 'Tscore_Femur_Neck', 'Zscore_Femur_neck', 'BMI', 'Active_Smoking' |
| 5 | RFECV (Logistic regression) | 15 | 'Tscore_Hip_Total', 'Cr', 'BMD_vertebra', 'Tscore_vertebra', 'Zscore_vertebra', 'BMD_Hip_Total', 'Zscore_Hip_Total', 'BMD_Femur_Neck', 'Tscore_Femur_Neck', 'Zscore_Femur_Neck', 'Histroy_Anticoagulant', 'Active_Smoking', 'History_Smoking', 'Calcium_Supplement', '‘History_of_diabetes’' |
| 6 | RFECV (LightGBM) | 14 | 'Tscore_Hip_total', 'CRP', 'Cr', 'ALP', 'BUN', 'P', 'Ca', 'PTH', 'Vit_D3', 'Tscore_ Spine', 'Zscore_ Spine', 'BMD_Hip_Total', 'Tscore_Femur_Neck', 'BMI' |
| 7 | RFECV (AdaBoost) | 2 | 'Cr', 'ALP' |

Table S3 Performance of female patients' prediction models on feature set 1

| **Algorithm** | **Parameters** | **Accuracy** |  | **AUC** | **Precision** | **Recall** | **F1 Score** | **Log Loss** | **Brier Score** |
| --- | --- | --- | --- | --- | --- | --- | --- | --- | --- |
| XGBoost | 'colsample_bytree': 0.3, 'learning_rate': 0.001, 'max_depth': 15,  'n_estimators': 700 | 0.807 |  | 0.888 | 0.793 | 0.838 | 0.813 | 0.604 | 0.206 |
| CatBoost | depth = 10,  l2_leaf_reg = 1,  learning_rate = 0.03,  n_estimators = 700 | 0.857 |  | 0.951 | 0.804 | 0.948 | 0.869 | 0.333 | 0.104 |
| Random Forest | 'max_depth': 32, 'min_samples_leaf': 3, 'min_samples_split': 8, 'n_estimators': 100 | 0.790 |  | 0.867 | 0.765 | 0.844 | 0.800 | 0.518 | 0.169 |
| LightGBM | 'boosting_type': 'gbdt', 'learning_rate': 0.1, 'n_estimators': 900,  'num_leaves': 12 | 0.800 |  | 0.904 | 0.754 | 0.899 | 0.818 | 0.688 | 160 |
| Logistic regression | 'C': 1000.0,  'penalty': 'l1',  'solver': 'liblinear' | 0.579 |  | 0.600 | 0.573 | 0.625 | 0.595 | 0.713 | 0.254 |
| ADABoost | 'learning_rate': 0.1,  'n_estimators': 900 | 0.633 |  | 0.652 | 0.615 | 0.717 | 0.660 | 0.691 | 0.249 |
| SVM | 'C': 100,  'gamma': 1,  'kernel': 'poly' | 0.643 |  | 0.667 | 0.627 | 0.708 | 0.662 | 0.665 | 0.233 |
| MLP | 'activation': 'tanh',  'alpha': 0.05, 'hidden_layer_sizes': (15, 10),  'learning_rate': 'constant',  'solver': 'lbfgs' | 0.719 |  | 0.731 | 0.684 | 0.813 | 0.741 | 2.318 | 0.245 |

Table S4 Performance of female patients' prediction models on feature set 2

| **Algorithm** | **Parameters** | **Accuracy** | **AUC** | **Precision** | **Recall** | **F1 Score** | **Log Loss** | **Brier Score** |
| --- | --- | --- | --- | --- | --- | --- | --- | --- |
| XGBoost | 'colsample_bytree': 0.8, 'learning_rate': 0.1,  'max_depth': 8,  'n_estimators': 500 | 0.823 | 0.905 | 0.782 | 0.908 | 0.839 | 0.445 | 0.135 |
| CatBoost | depth = 10,  l2_leaf_reg = 7,  learning_rate = 0.1,  n_estimators = 700 | 0.870 | 0.951 | 0.826 | 0.948 | 0.882 | 0.324 | 0.100 |
| Random Forest | 'max_depth': 16, 'min_samples_leaf': 3, 'min_samples_split': 8, 'n_estimators': 900 | 0.786 | 0.883 | 0.759 | 0.854 | 0.802 | 0.509 | 0.165 |
| LightGBM | 'boosting_type': 'gbdt', 'learning_rate': 0.1, 'n_estimators': 900,  'num_leaves': 8 | 0.813 | 0.898 | 0.765 | 0.911 | 0.831 | 0.558 | 0.149 |
| Logistic regression | 'C': 1.0,  'penalty': 'l2',  'solver': 'liblinear' | 0.577 | 0.585 | 0.590 | 0.524 | 0.553 | 0.686 | 0.247 |
| ADABoost | 'learning_rate': 0.1,  'n_estimators': 900 | 0.621 | 0.645 | 0.610 | 0.707 | 0.653 | 0.691 | 0.249 |
| SVM | 'C': 100,  'gamma': 1,  'kernel': 'poly' | 0.580 | 0.613 | 0.580 | 0.623 | 0.598 | 0.680 | 0.243 |
| MLP | 'activation': 'tanh',  'alpha': 0.05, 'hidden_layer_sizes': (15, 10),  'learning_rate': 'constant',  'solver': 'lbfgs' | 0.763 | 0.804 | 0.727 | 0.840 | 0.778 | 1.024 | 0.196 |

Table S5 Performance of female patients' prediction models on feature set 3

| **Algorithm** | **Parameters** | **Accuracy** | **AUC** | **Precision** | **Recall** | **F1 Score** | **Log Loss** | **Brier Score** |
| --- | --- | --- | --- | --- | --- | --- | --- | --- |
| XGBoost | 'colsample_bytree': 0.8, 'learning_rate': 0.1,  'max_depth': 15,  'n_estimators': 300 | 0.818 | 0.891 | 0.784 | 0.886 | 0.830 | 0.460 | 0.139 |
| CatBoost | depth = 10,  l2_leaf_reg = 1,  learning_rate = 0.03,  n_estimators = 600 | 0.853 | 0.940 | 0.805 | 0.936 | 0.865 | 0.354 | 0.111 |
| Random Forest | 'max_depth': 32, 'min_samples_leaf': 3, 'min_samples_split': 8, 'n_estimators': 300 | 0.792 | 0.862 | 0.768 | 0.847 | 0.803 | 0.521 | 0.170 |
| LightGBM | 'boosting_type': 'gbdt', 'learning_rate': 0.1, 'n_estimators': 900,  'num_leaves': 16 | 0.798 | 0.888 | 0.759 | 0.883 | 0.814 | 0.868 | 0.171 |
| Logistic regression | 'C': 10.0,  'penalty': 'l2',  'solver': 'newton-cg' | 0.618 | 0.661 | 0.608 | 0.652 | 0.628 | 0.664 | 0.235 |
| ADABoost | learning_rate= 0.1,  n_estimators= 900, | 0.640 | 0.665 | 0.626 | 0.716 | 0.665 | 0.691 | 0.249 |
| SVM | 'C': 100,  'gamma': 1,  'kernel': 'rbf' | 0.657 | 0.686 | 0.652 | 0.673 | 0.661 | 0.649 | 0.227 |
| MLP | 'activation': 'tanh',  'alpha': 0.05, 'hidden_layer_sizes': (15, 10),  'learning_rate': 'constant',  'solver': 'lbfgs' | 0.700 | 0.724 | 0.667 | 0.799 | 0.726 | 1.553 | 0.245 |

Table S6 Performance of female patients' prediction models on feature set 4

| **Algorithm** | **Parameters** | **Accuracy** | **AUC** | **Precision** | **Recall** | **F1 Score** | **Log Loss** | **Brier Score** |
| --- | --- | --- | --- | --- | --- | --- | --- | --- |
| XGBoost | 'colsample_bytree': 0.3, 'learning_rate': 0.1,  'max_depth': 15,  'n_estimators': 100 | 0.822 | 0.906 | 0.795 | 0.877 | 0.833 | 0.406 | 0.130 |
| CatBoost | depth = 10,  l2_leaf_reg = 1,  learning_rate = 0.1,  n_estimators = 600 | 0.868 | 0.956 | 0.819 | 0.950 | 0.879 | 0.322 | 0.100 |
| Random Forest | 'max_depth': 32, 'min_samples_leaf': 3, 'min_samples_split': 10, 'n_estimators': 100 | 0.786 | 0.866 | 0.764 | 0.844 | 0.800 | 0.522 | 0.171 |
| LightGBM | boosting_type': 'gbdt', 'learning_rate': 0.1, 'n_estimators': 700,  'num_leaves': 16 | 0.815 | 0.918 | 0.762 | 0.929 | 0.836 | 0.678 | 0.154 |
| Logistic regression | 'C': 1.0,  'penalty': 'l2',  'solver': 'liblinear' | 0.577 | 0.585 | 0.590 | 0.524 | 0.553 | 0.686 | 0.247 |
| ADABoost | 'learning_rate': 0.1  'n_estimators': 900 | 0.623 | 0.648 | 0.614 | 0.703 | 0.653 | 0.691 | 0.249 |
| SVM | 'C': 100,  'gamma': 1,  'kernel': 'poly' | 0.582 | 0.613 | 0.581 | 0.625 | 0.600 | 0.680 | 0.243 |
| MLP | 'activation': 'tanh',  'alpha': 0.05, 'hidden_layer_sizes': (15, 10),  'learning_rate': 'constant',  'solver': 'lbfgs' | 0.797 | 0.815 | 0.753 | 0.888 | 0.814 | 1.097 | 0.177 |

Table S7 Performance of female patients' prediction models on feature set 5

| **Algorithm** | **Parameters** | **Accuracy** | **AUC** | **Precision** | **Recall** | **F1 Score** | **Log Loss** | **Brier Score** |
| --- | --- | --- | --- | --- | --- | --- | --- | --- |
| XGBoost | 'colsample_bytree': 0.8, 'learning_rate': 0.01,  'max_depth': 6,  'n_estimators': 900 | 0.607 | 0.645 | 0.603 | 0.636 | 0.618 | 0.679 | 0.240 |
| CatBoost | depth = 4,  l2_leaf_reg = 1,  learning_rate = 0.03,  n_estimators = 700 | 0.619 | 0.658 | 0.610 | 0.663 | 0.633 | 0.668 | 0.235 |
| Random Forest | 'max_depth': 32, 'min_samples_leaf': 4, 'min_samples_split': 10, 'n_estimators': 100 | 0.599 | 0.641 | 0.599 | 0.618 | 0.607 | 0.667 | 0.237 |
| LightGBM | 'boosting_type': 'dart', 'learning_rate': 0.001, 'n_estimators': 100,  'num_leaves': 6 | 0.603 | 0.627 | 0.573 | 0.808 | 0.670 | 0.690 | 0.249 |
| Logistic regression | 'C': 1000.0,  'penalty': 'l1',  'solver': 'liblinear' | 0.580 | 0.623 | 0.570 | 0.659 | 0.609 | 0.675 | 0.241 |
| ADABoost | 'learning_rate': 0.1  'n_estimators': 900 | 0.593 | 0.620 | 0.583 | 0.655 | 0.616 | 0.692 | 0.250 |
| SVM | 'C': 0.1,  'gamma': 1,  'kernel': 'rbf' | 0.554 | 0.601 | 0.531 | 0.936 | 0.677 | 0.681 | 0.244 |
| MLP | 'activation': 'relu',  'alpha': 0.0001, 'hidden_layer_sizes': 10,  'learning_rate': 'constant',  'solver': 'lbfgs' | 0.574 | 0.620 | 0.559 | 0.751 | 0.632 | 0.677 | 0.242 |

Table S8 Performance of female patients' prediction models on feature set 6

| **Algorithm** | **Parameters** | **Accuracy** | **AUC** | **Precision** | **Recall** | **F1 Score** | **Log Loss** | **Brier Score** |
| --- | --- | --- | --- | --- | --- | --- | --- | --- |
| XGBoost | 'colsample_bytree': 0.8, 'learning_rate': 0.01,  'max_depth': 15,  'n_estimators': 700 | 0.769 | 0.843 | 0.756 | 0.798 | 0.775 | 0.496 | 0.162 |
| CatBoost | depth = 10,  l2_leaf_reg = 1,  learning_rate = 0.1,  n_estimators = 300 | 0.828 | 0.903 | 0.795 | 0.886 | 0.837 | 0.431 | 0.131 |
| Random Forest | 'max_depth': 16, 'min_samples_leaf': 3, 'min_samples_split': 8, 'n_estimators': 900 | 0.760 | 0.835 | 0.745 | 0.794 | 0.767 | 0.536 | 0.178 |
| LightGBM | 'boosting_type': 'gbdt', 'learning_rate': 0.1, 'n_estimators': 700,  'num_leaves': 16 | 0.760 | 0.828 | 0.730 | 0.826 | 0.774 | 0.761 | 0.190 |
| Logistic regression | 'C': 1000.0,  'penalty': 'l2',  'solver': 'newton-cg' | 0.557 | 0.568 | 0.562 | 0.531 | 0.545 | 0.691 | 0.248 |
| ADABoost | 'learning_rate': 0.1, 'n_estimators': 900 | 0.605 | 0.647 | 0.601 | 0.629 | 0.614 | 0.692 | 0.249 |
| SVM | 'C': 100,  'gamma': 1,  'kernel': 'rbf' | 0.570 | 0.587 | 0.581 | 0.539 | 0.554 | 0.687 | 0.247 |
| MLP | 'activation': 'tanh',  'alpha': 0.05, 'hidden_layer_sizes': (15, 10),  'learning_rate': 'constant',  'solver': 'lbfgs' | 0.678 | 0.715 | 0.669 | 0.709 | 0.687 | 0.741 | 0.228 |

Table S9 Performance of female patients' prediction models on feature set 7

| **Algorithm** | **Parameters** | **Accuracy** | **AUC** | **Precision** | **Recall** | **F1 Score** | **Log Loss** | **Brier Score** |
| --- | --- | --- | --- | --- | --- | --- | --- | --- |
| XGBoost | 'colsample_bytree': 0.8, 'learning_rate': 0.1,  'max_depth': 15,  'n_estimators': 700 | 0.764 | 0.841 | 0.729 | 0.845 | 0.782 | 0.617 | 0.181 |
| CatBoost | depth = 10,  l2_leaf_reg = 1,  learning_rate = 0.03,  n_estimators = 600 | 0.801 | 0.897 | 0.757 | 0.899 | 0.820 | 0.453 | 0.143 |
| Random Forest | 'max_depth': 32, 'min_samples_leaf': 3, 'min_samples_split': 8, 'n_estimators': 900 | 0.741 | 0.812 | 0.712 | 0.830 | 0.764 | 0.564 | 0.189 |
| LightGBM | 'boosting_type': 'gbdt', 'learning_rate': 0.1, 'n_estimators': 500,  'num_leaves': 16 | 0.758 | 0.833 | 0.727 | 0.837 | 0.776 | 0.721 | 0.194 |
| Logistic regression | 'C': 1.0,  'penalty': 'l1',  'solver': 'liblinear' | 0.583 | 0.594 | 0.593 | 0.540 | 0.562 | 0.684 | 0.245 |
| ADABoost | 'learning_rate': 0.1,  'n_estimators': 900 | 0.606 | 0.631 | 0.599 | 0.682 | 0.635 | 0.692 | 0.249 |
| SVM | 'C': 100,  'gamma': 0.01,  'kernel': 'sigmoid' | 0.566 | 0.581 | 0.635 | 0.322 | 0.422 | 0.690 | 0.249 |
| MLP | 'activation': 'tanh',  'alpha': 0.05, 'hidden_layer_sizes': (15, 10, 5),  'learning_rate': 'constant',  'solver': 'lbfgs' | 0.680 | 0.734 | 0.653 | 0.773 | 0.707 | 0.833 | 0.230 |

Table S10 Performance of male patients' prediction models on feature set 1

| **Algorithm** | **Parameters** | **Accuracy** | **AUC** | **Precision** | **Recall** | **F1 Score** | **Log Loss** | **Brier Score** |
| --- | --- | --- | --- | --- | --- | --- | --- | --- |
| XGBoost | n_estimators=900,  max_depth = 15,  learning_rate = 0.01, colsample_bytree = 0.3 | 0.920 | 0.973 | 0.893 | 0.953 | 0.921 | 0.234 | 0.066 |
| CatBoost | n_estimators = 300,  depth =10,  l2_leaf_reg = 1,  learning_rate = 0.03 | 0.928 | 0.987 | 0.885 | 0.985 | 0.932 | 0.202 | 0.058 |
| Random Forest | max_depth = 32, min_samples_leaf = 3,  min_samples_split = 8,  n_estimators = 100 | 0.885 | 0.955 | 0.867 | 0.909 | 0.886 | 0.383 | 0.112 |
| LightGBM | boosting_type = 'gbdt', learning_rate = 0.1,  n_estimators= 900,  num_leaves= 12, | 0.910 | 0.975 | 0.870 | 0.962 | 0.914 | 0.345 | 0.076 |
| Logistic regression | C = 1000,  penalty = 'l1',  solver = 'liblinear' | 0.690 | 0.716 | 0.676 | 0.748 | 0.705 | 0.897 | 0.228 |
| ADABoost | learning_rate= 0.1,  n_estimators= 900, | 0.784 | 0.833 | 0.744 | 0.874 | 0.797 | 0.683 | 0.245 |
| SVM | 'C': 100,  'gamma': 1,  'kernel': 'poly' | 0.746 | 0.770 | 0.696 | 0.877 | 0.775 | 0.633 | 0.201 |
| MLP | activation': 'tanh',  'alpha': 0.05,  'hidden_layer_sizes': (15, 10), 'learning_rate': 'constant',  'solver': 'lbfgs' | 0.825 | 0.830 | 0.780 | 0.912 | 0.839 | 1.210 | 0.160 |

Table S11 Performance of male patients' prediction models on feature set 2

| **Algorithm** | **Parameters** | **Accuracy** | **AUC** | **Precision** | **Recall** | **F1 Score** | **Log Loss** | **Brier Score** |
| --- | --- | --- | --- | --- | --- | --- | --- | --- |
| XGBoost | n_estimators=700,  max_depth = 15,  learning_rate = 0.01,  colsample_bytree = 0.3 | 0.923 | 0.975 | 0.911 | 0.938 | 0.923 | 0.270 | 0.074 |
| CatBoost | n_estimators = 300,  depth =6,  l2_leaf_reg = 1,  learning_rate = 0.1 | 0.931 | 0.984 | 0.888 | 0.988 | 0.935 | 0.197 | 0.055 |
| Random Forest | max_depth = 32,  min_samples_leaf = 3,  min_samples_split = 8,  n_estimators = 700 | 0.895 | 0.961 | 0.870 | 0.933 | 0.898 | 0.374 | 0.109 |
| LightGBM | boosting_type = 'dart',  learning_rate = 0.1,  n_estimators= 700,  num_leaves= 16, | 0.901 | 0.965 | 0.866 | 0.950 | 0.905 | 0.273 | 0.073 |
| Logistic regression | C = 1,  penalty = 'l2',  solver = 'newton-cg' | 0.624 | 0.641 | 0.620 | 0.646 | 0.631 | 0.679 | 0.243 |
| ADABoost | learning_rate= 0.1,  n_estimators= 500, | 0.766 | 0.806 | 0.31 | 0.842 | 0.781 | 0.679 | 0.243 |
| SVM | 'C': 100,  'gamma': 1,  'kernel': 'poly' | 0.674 | 0.722 | 0.642 | 0.787 | 0.705 | 0.618 | 0.212 |
| MLP | 'activation': 'tanh',  'alpha': 0.05,  'hidden_layer_sizes': (15, 10), 'learning_rate': 'constant',  'solver': 'lbfgs' | 0.863 | 0.906 | 0.809 | 0.956 | 0.876 | 0.616 | 0.112 |

Table S12 Performance of male patients' prediction models on feature set 3

| **Algorithm** | **Parameters** | **Accuracy** | **AUC** | **Precision** | **Recall** | **F1 Score** | **Log Loss** | **Brier Score** |
| --- | --- | --- | --- | --- | --- | --- | --- | --- |
| XGBoost | n_estimators=900,  max_depth = 15,  learning_rate = 0.01, colsample_bytree = 0.3 | 0.920 | 0.973 | 0.895 | 0.953 | 0.921 | 0.242 | 0.068 |
| CatBoost | n_estimators = 300,  depth =6,  l2_leaf_reg = 1,  learning_rate = 0.1 | 0.921 | 0.986 | 0.874 | 0.985 | 0.926 | 0.196 | 0.058 |
| Random Forest | max_depth = 16,  min_samples_leaf = 3,  min_samples_split = 8,  n_estimators = 300 | 0.889 | 0.955 | 0.876 | 0.909 | 0.890 | 0.383 | 0.112 |
| LightGBM | boosting_type = 'gbdt',  learning_rate = 0.1,  n_estimators= 900,  num_leaves= 8, | 0.914 | 0.968 | 0.884 | 0.953 | 0.917 | 0.399 | 0.074 |
| Logistic regression | C = 1000,  penalty = 'l1',  solver = 'liblinear' | 0.697 | 0.731 | 0.689 | 0.745 | 0.709 | 0.880 | 0.222 |
| ADABoost | learning_rate= 0.1,  n_estimators= 700, | 0.778 | 0.832 | 0.740 | 0.865 | 0.791 | 0.680 | 0.244 |
| SVM | 'C': 100,  'gamma': 1,  'kernel': 'poly' | 0.741 | 0.778 | 0.694 | 0.862 | 0.768 | 0.667 | 0.201 |
| MLP | 'activation': 'tanh',  'alpha': 0.05,  'hidden_layer_sizes': (15, 10), 'learning_rate': 'constant',  'solver': 'lbfgs' | 0.805 | 0.812 | 0.762 | 0.889 | 0.819 | 1.417 | 0.179 |

Table S13 Performance of male patients' prediction models on feature set 4

| **Algorithm** | **Parameters** | **Accuracy** | **AUC** | **Precision** | **Recall** | **F1 Score** | **Log Loss** | **Brier Score** |
| --- | --- | --- | --- | --- | --- | --- | --- | --- |
| XGBoost | n_estimators=900,  max_depth = 15,  learning_rate = 0.01, colsample_bytree = 0.5 | 0.857 | 0.922 | 0.830 | 0.903 | 0.863 | 0.358 | 0.111 |
| CatBoost | n_estimators = 700,  depth =10,  l2_leaf_reg = 3,  learning_rate = 0.03 | 0.877 | 0.955 | 0.837 | 0.945 | 0.886 | 0.307 | 0.094 |
| Random Forest | max_depth = 32,  min_samples_leaf = 3,  min_samples_split = 10,  n_estimators = 300 | 0.825 | 0.903 | 0.804 | 0.862 | 0.828 | 0.445 | 0.140 |
| LightGBM | boosting_type = 'gbdt',  learning_rate = 0.1,  n_estimators= 900,  num_leaves= 12, | 0.863 | 0.924 | 0.835 | 0.912 | 0.869 | 0.760 | 0.121 |
| Logistic regression | C = 1000,  penalty = 'l1',  solver = 'liblinear' | 0.697 | 0.721 | 0.678 | 0.757 | 0.708 | 0.881 | 0.225 |
| ADABoost | learning_rate= 0.1,  n_estimators= 900 | 0.750 | 0.789 | 0.732 | 0.804 | 0.761 | 0.688 | 0.247 |
| SVM | 'C': 0.1,  'gamma': 1,  'kernel': 'rbf' | 0.674 | 0.683 | 0.633 | 0.783 | 0.697 | 0.647 | 0.226 |
| MLP | 'activation': 'tanh',  'alpha': 0.05,  'hidden_layer_sizes': (10, 5), 'learning_rate': 'constant',  'solver': 'lbfgs' | 0.738 | 0.758 | 0.720 | 0.807 | 0.755 | 1.353 | 0.206 |

Table S14 Performance of male patients' prediction models on feature set 5

| **Algorithm** | **Parameters** | **Accuracy** | **AUC** | **Precision** | **Recall** | **F1 Score** | **Log Loss** | **Brier Score** |
| --- | --- | --- | --- | --- | --- | --- | --- | --- |
| XGBoost | n_estimators=900,  max_depth = 15,  learning_rate = 0.01, colsample_bytree = 0.5 | 0.857 | 0.922 | 0.830 | 0.903 | 0.863 | 0.358 | 0.111 |
| CatBoost | n_estimators = 700,  depth =10,  l2_leaf_reg = 3,  learning_rate = 0.03 | 0.877 | 0.955 | 0.837 | 0.945 | 0.886 | 0.307 | 0.094 |
| Random Forest | max_depth = 32,  min_samples_leaf = 3,  min_samples_split = 10,  n_estimators = 100 | 0.839 | 0.900 | 0.817 | 0.883 | 0.844 | 0.447 | 0.140 |
| LightGBM | boosting_type = 'gbdt',  learning_rate = 0.1,  n_estimators= 900,  num_leaves= 12, | 0.863 | 0.924 | 0.835 | 0.912 | 0.869 | 0.760 | 0.121 |
| Logistic regression | C = 1000,  penalty = 'l1',  solver = 'liblinear' | 0.697 | 0.721 | 0.678 | 0.757 | 0.708 | 0.881 | 0.225 |
| ADABoost | learning_rate= 0.1,  n_estimators= 900 | 0.750 | 0.789 | 0.732 | 0.804 | 0.761 | 0.688 | 0.247 |
| SVM | 'C': 0.1,  'gamma': 1,  'kernel': 'rbf' | 0.674 | 0.683 | 0.633 | 0.783 | 0.697 | 0.647 | 0.226 |
| MLP | 'activation': 'tanh',  'alpha': 0.05,  'hidden_layer_sizes': (10, 5), 'learning_rate': 'constant',  'solver': 'lbfgs' | 0.738 | 0.758 | 0.720 | 0.807 | 0.755 | 1.353 | 0.206 |

Table S15 Performance of male patients' prediction models on feature set 6

| **Algorithm** | **Parameters** | **Accuracy** | **AUC** | **Precision** | **Recall** | **F1 Score** | **Log Loss** | **Brier Score** |
| --- | --- | --- | --- | --- | --- | --- | --- | --- |
| XGBoost | n_estimators=900,  max_depth = 4,  learning_rate = 0.1, colsample_bytree = 0.8 | 0.909 | 0.963 | 0.871 | 0.965 | 0.915 | 0.264 | 0.075 |
| CatBoost | n_estimators = 600,  depth =10,  l2_leaf_reg = 1,  learning_rate = 0.03 | 0.934 | 0.990 | 0.895 | 0.985 | 0.938 | 0.175 | 0.051 |
| Random Forest | max_depth = 16,  min_samples_leaf = 3,  min_samples_split = 8,  n_estimators = 100 | 0.888 | 0.955 | 0.861 | 0.927 | 0.892 | 0.395 | 0.117 |
| LightGBM | boosting_type = 'gbdt',  learning_rate = 0.1,  n_estimators= 900,  num_leaves= 16, | 0.909 | 0.968 | 0.864 | 0.974 | 0.915 | 0.431 | 0.077 |
| Logistic regression | C = 1000,  penalty = 'l1',  solver = 'liblinear' | 0.589 | 0.617 | 0.582 | 0.653 | 0.611 | 0.689 | 0.245 |
| ADABoost | learning_rate= 0.1,  n_estimators= 900 | 0.755 | 0.802 | 0.719 | 0.833 | 0.771 | 0.684 | 0.245 |
| SVM | 'C': 100,  'gamma': 1,  'kernel': 'rbf' | 0.630 | 0.678 | 0.625 | 0.673 | 0.644 | 0.659 | 0.231 |
| MLP | 'activation': 'tanh',  'alpha': 0.05,  'hidden_layer_sizes': (15, 10), 'learning_rate': 'constant',  'solver': 'lbfgs' | 0.864 | 0.890 | 0.807 | 0.962 | 0.877 | 0.631 | 0.120 |

Table S16 Performance of male patients' prediction models on feature set 7

| **Algorithm** | **Parameters** | **Accuracy** | **AUC** | **Precision** | **Recall** | **F1 Score** | **Log Loss** | **Brier Score** |
| --- | --- | --- | --- | --- | --- | --- | --- | --- |
| XGBoost | n_estimators=500,  max_depth = 3,  learning_rate = 0.01, colsample_bytree = 0.3 | 0.589 | 0.597 | 0.587 | 0.628 | 0.604 | 0.688 | 0.246 |
| CatBoost | n_estimators = 300,  depth =10,  l2_leaf_reg = 7,  learning_rate = 0.03 | 0.715 | 0.779 | 0.695 | 0.778 | 0.731 | 0.568 | 0.192 |
| Random Forest | max_depth = 16,  min_samples_leaf = 3,  min_samples_split = 8,  n_estimators = 300 | 0.697 | 0.747 | 0.692 | 0.728 | 0.706 | 0.601 | 0.205 |
| LightGBM | boosting_type = 'dart',  learning_rate = 0.1,  n_estimators= 100,  num_leaves= 16, | 0.696 | 0.727 | 0.694 | 0.716 | 0.700 | 0.614 | 0.211 |
| Logistic regression | C = 1000,  penalty = 'l1',  solver = 'liblinear' | 0.563 | 0.580 | 0.572 | 0.549 | 0.556 | 0.688 | 0.247 |
| ADABoost | learning_rate= 0.1,  n_estimators= 900 | 0.577 | 0.602 | 0.570 | 0.643 | 0.603 | 0.692 | 0.250 |
| SVM | 'C': 100,  'gamma': 1,  'kernel': 'rbf' | 0.509 | 0.520 | 0.506 | 0.520 | 0.510 | 0.696 | 0.251 |
| MLP | 'activation': 'tanh',  'alpha': 0.0001, 'hidden_layer_sizes': 15, 'learning_rate': 'constant',  'solver': 'lbfgs' | 0.550 | 0.575 | 0.545 | 0.568 | 0.555 | 0.772 | 0.250 |
