# Supplementary material for: Prediction of subsequent fragility fractures: application of machine learning
Source: BMC Musculoskelet Disord. 2024 Jun 4;25:438. doi: 10.1186/s12891-024-07559-y (PMC11149176; doi:10.1186/s12891-024-07559-y)
Supplement: Supplementary file 2 — Supplementary Material 2 [file 12891_2024_7559_MOESM2_ESM.docx]

**Table of Figures**

[Figure S1. Confusion matrix and heatmap of error cases in CatBoost prediction for female patients 2](#_Toc161332533)

[Figure S2. Confusion matrix and heatmap of error cases in CatBoost prediction for male patients 3](#_Toc161332534)


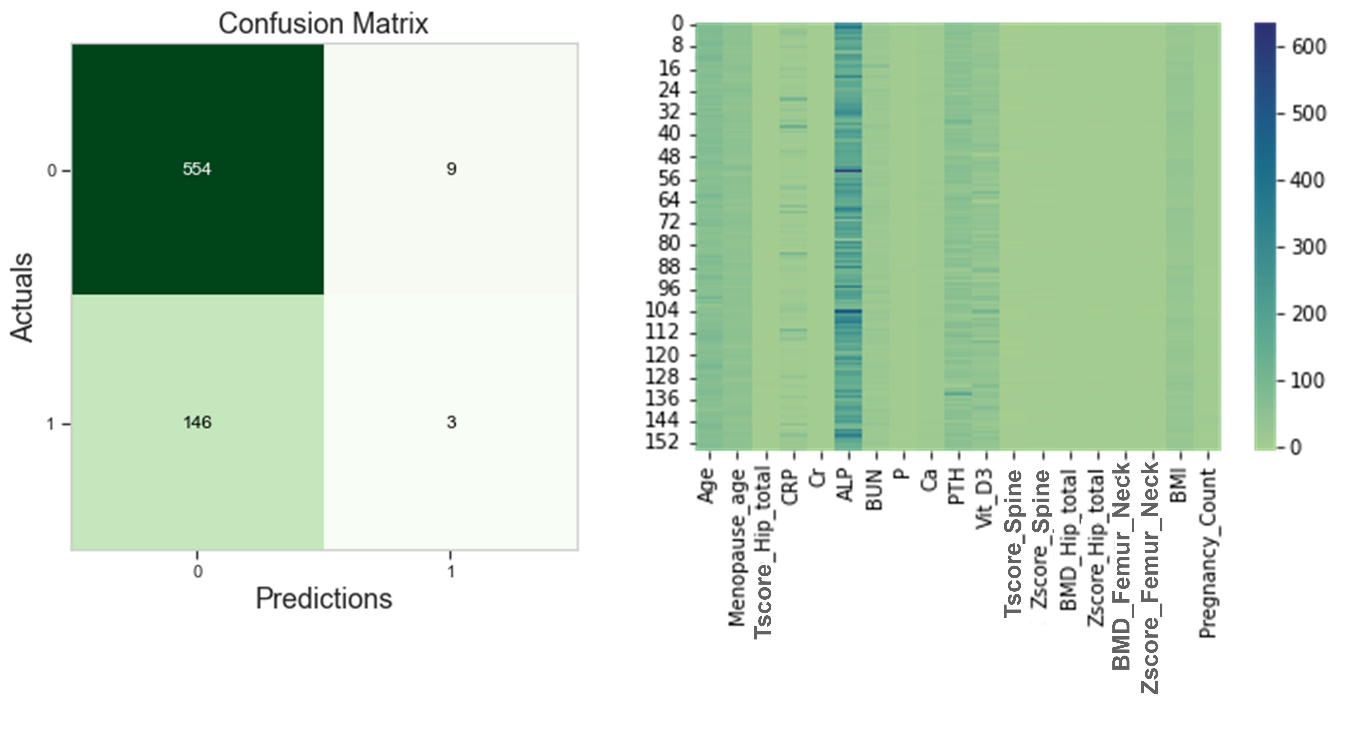


Figure S1. Confusion matrix and heatmap of error cases in CatBoost prediction for female patients


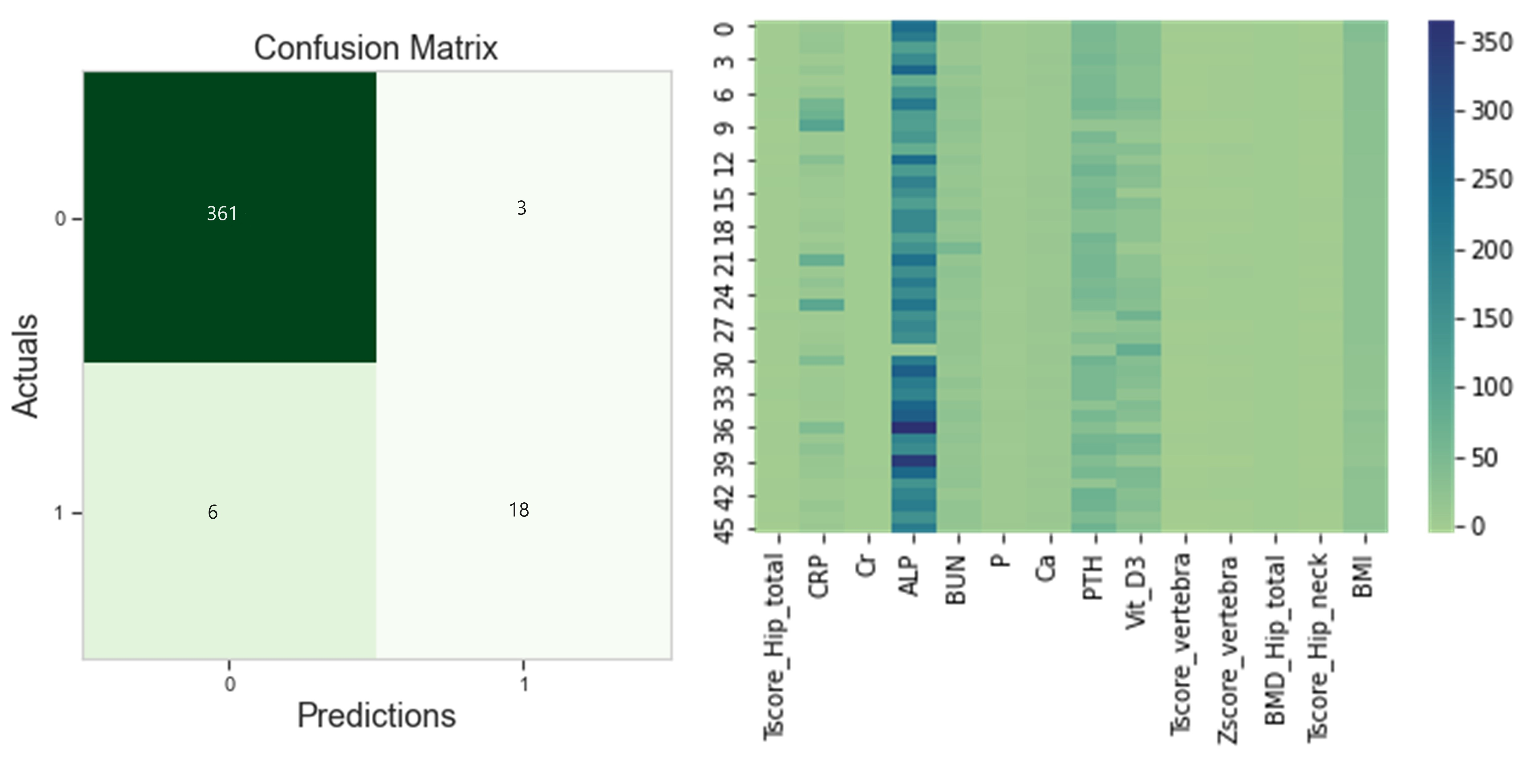


Figure S2. Confusion matrix and heatmap of error cases in CatBoost prediction for male patients
